# Catecholate Siderophores Protect Bacteria from Pyochelin Toxicity

**DOI:** 10.1371/journal.pone.0046754

**Published:** 2012-10-05

**Authors:** Conrado Adler, Natalia S. Corbalán, Mohammad R. Seyedsayamdost, María Fernanda Pomares, Ricardo E. de Cristóbal, Jon Clardy, Roberto Kolter, Paula A. Vincent

**Affiliations:** 1 Departamento de Bioquímica de la Nutrición, INSIBIO (Consejo Nacional de Investigaciones Científicas y Técnicas-Universidad Nacional de Tucumán) San Miguel de Tucumán, Tucumán, Argentina; 2 Department of Biological Chemistry and Molecular Pharmacology, Harvard Medical School, Boston, Massachusetts, United States of America; 3 Department of Microbiology and Immunobiology, Harvard Medical School, Boston, Massachusetts, United States of America; Auburn University, United States of America

## Abstract

**Background:**

Bacteria produce small molecule iron chelators, known as siderophores, to facilitate the acquisition of iron from the environment. The synthesis of more than one siderophore and the production of multiple siderophore uptake systems by a single bacterial species are common place. The selective advantages conferred by the multiplicity of siderophore synthesis remains poorly understood. However, there is growing evidence suggesting that siderophores may have other physiological roles besides their involvement in iron acquisition.

**Methods and Principal Findings:**

Here we provide the first report that pyochelin displays antibiotic activity against some bacterial strains. Observation of differential sensitivity to pyochelin against a panel of bacteria provided the first indications that catecholate siderophores, produced by some bacteria, may have roles other than iron acquisition. A pattern emerged where only those strains able to make catecholate-type siderophores were resistant to pyochelin. We were able to associate pyochelin resistance to catecholate production by showing that pyochelin-resistant *Escherichia coli* became sensitive when biosynthesis of its catecholate siderophore enterobactin was impaired. As expected, supplementation with enterobactin conferred pyochelin resistance to the *entE* mutant. We observed that pyochelin-induced growth inhibition was independent of iron availability and was prevented by addition of the reducing agent ascorbic acid or by anaerobic incubation. Addition of pyochelin to *E. coli* increased the levels of reactive oxygen species (ROS) while addition of ascorbic acid or enterobactin reduced them. In contrast, addition of the carboxylate-type siderophore, citrate, did not prevent pyochelin-induced ROS increases and their associated toxicity.

**Conclusions:**

We have shown that the catecholate siderophore enterobactin protects *E. coli* against the toxic effects of pyochelin by reducing ROS. Thus, it appears that catecholate siderophores can behave as protectors of oxidative stress. These results support the idea that siderophores can have physiological roles aside from those in iron acquisition.

## Introduction

In response to iron starvation, many bacteria synthesize and secrete siderophores, small molecules with high affinities for iron [1], [2]. While there is a very large number of different siderophores, they all belong to a few structural classes, including catecholate, carboxylate, hydroxamate, and mixed ligand siderophores [3]. Once ferrated, all siderophores - regardless of structural class - must be taken up into the cell, which is accomplished via dedicated transport systems [4]. Microbial cells are thus able to scavenge iron, usually present in short supply due to its low solubility. Interestingly, many microbes produce the transport systems for siderophores that they themselves do not synthesize, allowing for “siderophore piracy” [5].

The capacity to produce numerous iron uptake systems is exemplified by the enteric bacterium *Escherichia coli,* where the process has been extensively studied. *E. coli* produces its primary siderophore enterobactin and takes it up via the *fep* system [6]. In addition, *E. coli* strains may have up to eight other iron uptake systems including those for ferrichrome (*fhu*), ferric citrate (*fec*), aerobactin (*iut*), heme (*chu*), rhodotorulic acid and coprogen (*fhuE*), salmochelin (*iro*), yersiniabactin (*ybt*), and ferrous iron (*feo*) [7], [8], [9], [10], [11], [12], [13], [14], [15].

**Figure 1:**
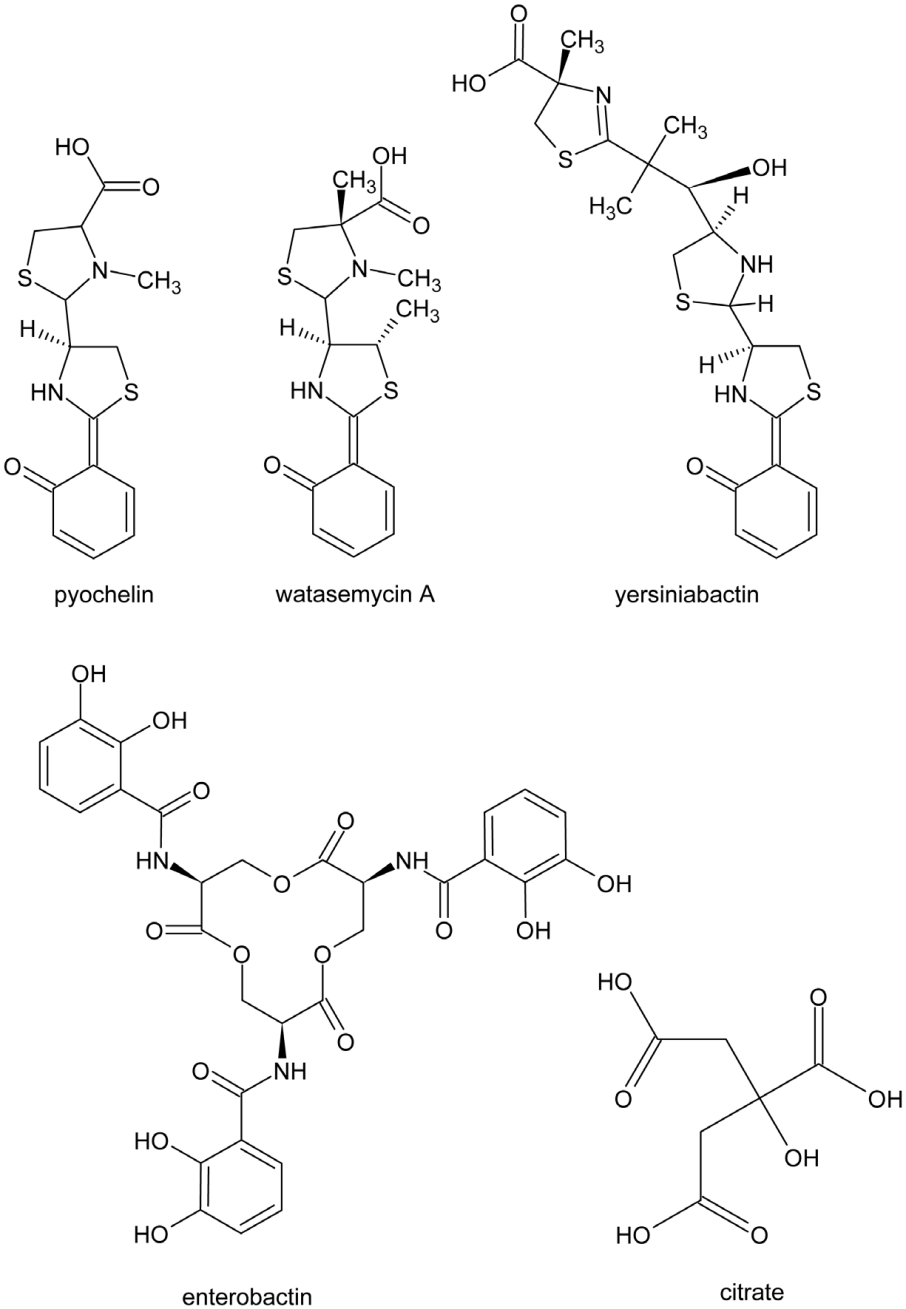
Chemical structures of the siderophores pyochelin, watasemycin A, yersiniabactin, enterobactin and citrate.

Aside from their ability to provide access to iron, siderophores may have alternative roles. A representative example is the production of pyochelin by the opportunistic pathogen *Pseudomonas aeruginosa.* This bacterium produces two siderophores: pyoverdine, with a high affinity for iron, and pyochelin, whose affinity for iron is much lower [16], [17]. Although *P. aeruginosa* can use pyochelin as a *bona fide* siderophore, pyochelin’s role remains unclear. Interestingly, pyochelin has been shown to have non-specific toxic effects on eukaryotic cells due to its ability to generate reactive oxygen species [18]. Watasemycins, molecules structurally related to pyochelin (Fig. 1), have been shown to have antibiotic activity as well [19]. There are previous reports showing antibiotic activity for other siderophores with varied chemical structures, e.g oxachelin [20], fusigen [21] and the sideromycins: albomycin and salmycin [22].

Here we report an interesting interaction between pyochelin and enterobactin producers that occurs independent of the availability of iron. While investigating bacterial interspecies interactions, we discovered that *P. aeruginosa* kills *Xanthomonas citri* subsp *citri in vitro.* We purified the compound responsible for the antibiotic activity and showed that it was pyochelin (Fig. 1). This came as a surprise as there had been no prior reports of pyochelin’s antibacterial activity. Analysis of the spectrum of the antibacterial activity of pyochelin revealed that bacteria unable to produce catecholate siderophores were sensitive to pyochelin while catecholate siderophore producers were resistant. We also show that pyochelin toxicity is not a result of iron competition, but due to the generation of reactive oxygen species (ROS), and that catechol siderophores confer resistance to pyochelin by reducing ROS. These results further solidify the observations that pyochelin is not just a siderophore but can also have toxic effects on diverse cells, and suggest that catecholate siderophores can play a role as protectors against oxidative stress.

## Results

### Pyochelin Inhibits Non Catechol-siderophore-producer Strains

Our initial interest was to identify bacteria capable of killing *Xanthomonas citri* subsp. *citri,* the causative agent of citrus canker disease. In a screen for bacteria that had anti-*Xanthomonas* activity, we discovered that strains of *P. aeruginosa* displayed antibiosis against *X. citri*. We used HPLC-MS and UV spectral analyses to characterize the compound responsible for this antibiotic activity and found it to be pyochelin (Fig. S1). We began to investigate the properties of pyochelin by exploring the spectrum of its antibiotic activity. We tested eleven bacterial strains for their resistance or sensitivity to pyochelin. Table 1 shows the minimal inhibitory concentrations (MIC) for the strains tested. Several *Xanthomonas* species and *Staphylococcus aureus* were sensitive. In contrast, several members of the *Enterobacteriaceae* were resistant. We thus became interested in determining the molecular basis for the differential sensitivity to pyochelin among different bacteria.

**Table 1:** MIC of pyochelin against selected bacterial strains.

Strain	MIC^a^
*Xanthomonas citri* subsp. *Citri*	48
*Xanthomonas campestris* pv *campestris*	24
*Xanthomonas campestris* pv *vesicatoria*	48
*Xanthomonas albilineans*	48
*Staphylococcus aureus* ATCC 25923	3
*Escherichia coli* ME9062 BW 25113	R
*Salmonella enterica* serovar Typhimurium 14028	R
*Klebsiella pneumoniae*	R
*Citrobacter freundii*	R
*Enterobacter cloacae*	R
*Serratia marcescens*	R

a MIC determination in M9 medium expressed in µM.

R = Resistant.

Our initial hypothesis was that the range of sensitivity to pyochelin was due to differences among these bacteria in their ability to acquire iron in the presence of pyochelin. To test this hypothesis we determined the MIC of pyochelin on the sensitive strains in the presence of excess of iron (100 µM). Surprisingly, we observed virtually no change in MIC (at most a one-fold dilution difference) when iron was added (Table 2). This allowed us to rule out iron sequestration as a mechanism for pyochelin toxicity. These results required the formulation of an alternative hypothesis.

**Table 2:** Influence of iron on the MIC of pyochelin.

Strain	MIC^a^	MIC + FeCl_3_ b
*Xanthomonas citri* subsp. *citri*	48	96
*Xanthomonas campestris* pv *campestris*	24	48
*Xanthomonas campestris* pv *vesicatoria*	48	96
*Xanthomonas albilineans*	48	96
*Staphylococcus aureus* ATCC 25923	3	3

MIC values are expressed in µM.

a MIC determination in M9 medium.

b MIC determination in M9 medium supplemented with 100 µM FeCl_3._

We reasoned that perhaps differences in siderophore production might underlie pyochelin resistance. A literature survey indicated that all resistant strains were able to synthesize catecholate siderophores. Specifically, all resistant strains were able to synthesize the tri-catecholate siderophore enterobactin. In contrast, sensitive strains were not. Instead, they produce other structural types of siderophores; *S. aureus* produces the carboxylate siderophore staphyloferrin A [23] while *Xanthomonas spp.* produce α-hydroxycarboxylate-type siderophores [24], [25].

These insights from the literature allowed us to refine our hypothesis that enterobactin was, in some way, responsible for pyochelin resistance. To test this hypothesis we focused our attention on the genetically tractable model bacterium *E. coli*. We compared the sensitivity to pyochelin using a wild-type *E. coli* and a mutant impaired in enterobactin synthesis (*E. coli entE*). Consistent with our hypothesis, *E. coli* unable to synthesize enterobactin were highly sensitive to pyochelin (Table 3). As was the case with the naturally sensitive strains, iron supplementation did not rescue *E. coli entE* from pyochelin toxicity (Table 3).

**Table 3:** MIC of pyochelin in the presence of selected additives.

	MIC				
Strain	M9^a^	ASC^b^	FeCl_3_ c	ENT^d^	Citrate^e^
*Escherichia coli* BW 25113	R	ND	ND	ND	ND
*Escherichia coli* JW0586-1 (*entE*)	12	192	24	R	12

MIC values are expressed in µM.

ND = Not Determined.

R = Resistant (no inhibition observed) to pyochelin at a concentration of 3 mM.

a M9 medium.

b M9 medium supplemented with 1 mM ascorbic acid.

c M9 medium supplemented with 100 µM FeCl_3_.

d M9 medium supplemented with 1 µM enterobactin.

e M9 medium supplemented with 50 µM citrate.

### Pyochelin Toxicity is Due to Generation of Reactive Oxygen Species (ROS)

**Figure 2:**
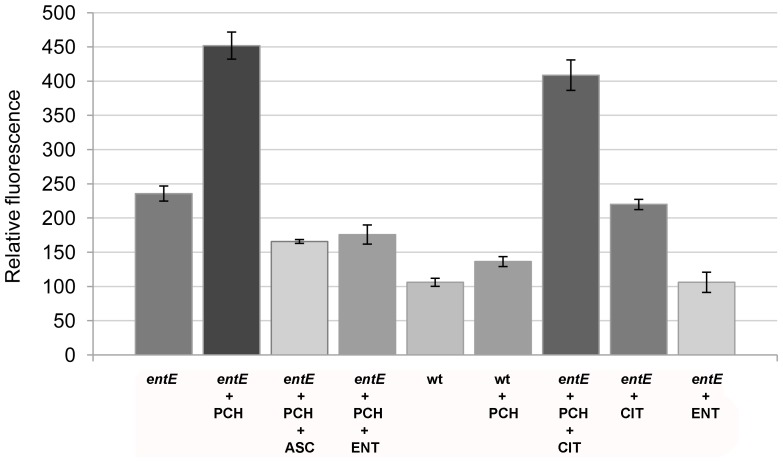
Levels of reactive oxygen species in *E. coli* wild type and *entE* mutant. Quantitation of ROS levels using the DCFA-DA probe. Fluorescence intensities are relative to that of the control. Control: wt grown in 2 mL M9 medium; PCH indicates cells grown in the presence of 15 µM pyochelin; ASC indicates supplementation with 1 mM ascorbic acid; ENT designates addition of 1 µM of pure enterobactin; CIT indicates supplementation with 50 µM citrate. Error bars = SEM, n = 3.

It has been previously reported that pyochelin can catalyse the Haber-Weiss reaction *in vitro* and therefore generate reactive oxygen species (ROS) [26], [27]. In addition, it has been shown that endothelial cells exposed to pyochelin and pyocyanin together suffered from cellular damage due to the generation of hydroxyl radicals [18]. Taking into account that the pyochelin-induced bacterial growth inhibition was not affected by iron concentrations, we hypothesized that pyochelin could be inhibiting bacterial growth of the sensitive strains by causing oxidative damage. Quantitation of ROS using DCFA-DA, a fluorescent reporter of ROS [28], revealed increased ROS levels in *E. coli entE* after exposure to pyochelin (Fig. 2). Importantly and as expected, addition of the reducing agent ascorbic acid resulted in lower ROS levels (Fig. 2). Consistent with the idea that ROS are the cause of pyochelin sensitivity, ascorbic acid reduced the senstivity of *E. coli entE* to pyochelin (Table 3). In addition, anaerobic culture conditions rendered *E. coli entE* less sensitive to pyochelin (Table 4). These results indicate a correlation between ROS levels and pyochelin toxicity.

**Table 4:** Growth inhibition by pyochelin under aerobic and anaerobic culture conditions.

	Aerobic		Anaerobic	
Strain	control	pyochelin	control	pyochelin
*Escherichia coli* JW0586-1 (*entE*)	100±5%	22±3%	100±8%	69±3%

Growth was determined by OD measurement after 20 hours of standing culture.

Values are relative to OD values for aerobic and anaerobic controls (no pyochelin addition).

SEM calculated from 3 different experiments.

### Enterobactin Reduces the Levels of ROS and Sensitivity to Pyochelin

Given that a mutation in the enterobactin biosynthetic pathway rendered *E. coli* sensitive to pyochelin (Table 3), and that pyochelin toxicity was correlated with ROS levels, we wondered if enterobactin could reduce them. Because of their low radical reduction potentials, catechols can act as hydrogen atom donors and efficiently terminate radical chain reactions [29]. Fig. 2 shows that 1 µM enterobactin prevented pyochelin-mediated ROS generation in an *entE* mutant. Accordingly, a wild-type *E. coli* strain showed a small increase in ROS levels upon pyochelin addition (Fig. 2). As expected, addition of 1 µM enterobactin rendered *E. coli entE* resistant to pyochelin (Table 3). In contrast, addition of the carboxylate-type siderophore, citrate (50 µM) (Fig. 1), had no effect on the pyochelin sensitivity of the *E. coli entE* strain (Table 3). Moreover, citrate addition (50 µM) did not reduce pyochelin-induced ROS (Fig. 2). In addition, comparison of ROS levels in wild-type *E. coli* and *E. coli entE* in the absence of pyochelin showed increased ROS levels in the strain impaired in enterobactin synthesis (Fig. 2). Finally, supplementing *E. coli entE* with 1 µM enterobactin lowered ROS to the levels observed in the wild type (Fig. 2). Given that enterobactin contains three catechol groups (Fig. 1) and lowers ROS levels, we suggest a role for enterobactin as a protector from the damaging effects of ROS.

## Discussion

The results presented in this work suggest two new activities for two old, known siderophores: First, that pyochelin acts as an antibacterial agent due to its capacity to generate ROS, and second, that enterobactin can protect against ROS. Bacterial species that produce catecholate siderophores such as enterobactin, are resistant to pyochelin while those that synthesize carboxylate-type siderophores are sensitive to it. Therefore, despite their ability to chelate and facillitate iron uptake, carboxylate siderophores do not have the capacity to protect cells from the toxic effects of pyochelin. Importantly, impairment of enterobactin synthesis by mutation rendered *E. coli* sensitive to pyochelin. The fact that sensitivity to pyochelin did not change by iron supplementation or by citrate addition further supports the protective role proposed for catechols. Given that both enterobactin and citrate facilitate iron uptake, we ruled out an enterobactin protection mechanism connected with simple iron acquisition. Then, we observed that pyochelin increased ROS levels in an *E. coli entE* culture and that addition of the reducing agent ascorbic acid or enterobactin counteracted this effect. In contrast, citrate did not reduce pyochelin-induced ROS. Based on iron affinity constants of enterobactin (10^49^ M^−1^) [4] and citrate (10^24^ M^−1^) [30], we reasoned that both siderophores would be able to displace iron from any pyochelin-iron complex (pyochelin Ka = 10^5^ M^−1^) [31]. However, since only enterobactin protects against pyochelin toxicity, we can assume that the enterobactin protection mechanism would not imply lessening the generation of pyochelin-induced ROS by sequestering iron. Therefore, we suggest that enterobactin protects against pyochelin-induced oxidative stress by a mechanism involving radical scavenging, as is reported for polyphenols [32]. The reduced toxicity of pyochelin in anaerobic culture conditions strenghthens our hypothesis. *In toto*, our results provide additional evidence suggesting that molecules with siderophore activity can play other roles in microbial physiology.

Several features of pyochelin’s chemistry and biology are indicative that its major role may not be that of an iron chelator. Pyochelin’s affinity for iron is low and it binds other metals, e.g. copper, with similar affinities [31]. Pyochelin also lacks six chelating groups to participate in iron complexation, a feature typical of many *bona fide* siderophores [16]. At the same time, pyochelin’s ability to generate ROS and its antibacterial activity point to its potential to act as a toxin. Along these lines, it was previously shown that pyochelin along with pyoverdine were in part responsible for the *P. aeruginosa-*mediated suppression of *Pythium*-induced damping-off of tomato [33]. In addition, watasemycins, compounds structurally close to pyochelin (Fig. 1), have been reported to have antibiotic activity against *S. aureus* but not against *E. coli* [19]. On the other hand, yersiniabactin, a siderophore produced by *Yersinia* species [34], *Pseudomonas syringae* [35], and some uropathogenic *E. coli* strains [36], even though it is structurally close to pyochelin (Fig. 1), did not inhibit *E. coli entE, X. citri* subsp. *citri* or *S. aureus* at concentrations up to 2 mM (Data not shown). This indicates that structural differences between pyochelin, watasemycins and yersiniabactin are relevant in terms of the role each siderophore may play, perhaps by affecting their ability to reach their celullar targets and/or by affecting ROS generation. Like pyochelin, the redox-active phenazines, also produced by *P. aeruginosa*, have been shown to have antimicrobial activity through ROS generation and they also facilitate iron uptake [37], [38]. The primary role of phenazines may be to participate in intercelullar signalling that leads to extracelullar matrix synthesis and the consequent development of complex colony architecture [39]. The possibility of pyochelin taking part in similar developmental processes should not be overlooked. It will thus be important to further examine other physiological roles of pyochelin and the benefits that it confers to producer strains.

Siderophore biosynthesis is primarily regulated by iron availability but there is evidence that their production can be influenced by other stimuli, particularly agents that mediate oxidative damage. Catecholate siderophore biosynthesis in *Azotobacter vinelandii* has been shown to be under SoxS control and responsive to oxidative stress [40], [41]. When treated with paraquat, *Bacillus anthracis* accumulates the two catecholate siderophores bacillibactin and petrobactin [42]. Interestingly, iron-mediated repression of petrobactin synthesis is delayed under highly aerated culture conditions [42]. These results emphasize the existence of factors involved in the regulation of siderophore biosynthesis in addition to the lack of iron availability. Since enterobactin protects against the ROS-mediated toxic effects of pyochelin and impairment of enterobactin biosynthesis increases ROS levels, we suggest that catechols may be employed as protectants against oxidative stress. Thus, enterobactin should be added to the long list of defences that *E. coli* has evolved to cope with conditions of oxidative stress.

## Materials and Methods

### Bacterial Strains and Growth Conditions

Strains used in this work are listed in Table 5. Strains were grown in M9 medium supplemented with 0.2% Casamino acids, 0.2% glucose, 1 mM MgSO_4_ and 1 µg/mL vitamin B1. Solid media contained 1.5% agar. Kanamycin 50 µg/mL, was added when required. Standing cultures were performed in 2 mL tubes containing 1.5 mL of culture medium. 300 µL of mineral oil was added for anaerobic cultures.

**Table 5:** List of strains used in this work.

Strain	Relevant genotype	Source
*Pseudomonas aeruginosa* PAO1	wild type	PAML^a^
*Xanthomonas citri* subsp. *citri*	wild type	EEAOC^b^
*Xanthomonas campestris* pv. *campestris*	wild type	EEAOC^b^
*Xanthomonas campestris* pv *vesicatoria*	wild type	EEAOC^b^
*Xanthomonas albilineans*	wild type	EEAOC^b^
*Staphylococcus aureus* ATCC 25923	wild type	ATCC^c^
*Escherichia coli* ME9062 BW 25113	wild type	CGSC^d^
*Escherichia coli* JW 0586-1	BW25113Δ*entE*::*kan*	CGSC^d^
*Salmonella enterica* serovar Typhimurium14028	wild type	UNT^e^
*Klebsiella pneumoniae*	wild type	UNT^e^
*Citrobacter freundii*	wild type	UNT^e^
*Enterobacter cloacae*	wild type	UNT^e^
*Serratia marcescens*	wild type	UNT^e^

a PAML, *Pseudomonas aeruginosa* Mutant Library;

b EEAOC, Estación Experimental Agroindustrial Obispo Colombres;

c ATCC, American Type Culture Collection;

d CGSC, *E. coli* Genetic Stock Center;

e UNT, Universidad Nacional de Tucumán- Cátedra de Bacteriología.

### Pyochelin Purification

Pyochelin was obtained from *Pseudomonas aeruginosa* PA01 cultures grown for 20 h at 30°C in M9 medium supplemented with 0.2% Casamino acids, 0.2% glucose, 1 mM MgSO_4_ and 1 µg/mL vitamin B1. The cell free supernatant was loaded into a 1 g C18 cartridge (Phenomenex) which had been equilibrated in 20% MeOH in water, and eluted stepwise with 40%, 60%, 80% and 100% methanol (in water). The 40% fraction was concentrated *in vacuo* and further purified by HPLC using a C18 Phenomenex Luna column (4.6×10 mm, 5 micron) and a gradient of 10 to 85% acetonitrile in water containing 0.1% trifluoracetic acid at a flow rate of 1 mL/min. Pyochelin I and II eluted at 42 and 46% MeCN, respectively. The structure of pyochelin was confirmed by HPLC-MS ([M+H]^+^exp 325.1, obs. 325.1 for both diastereomers) and UV spectral analyses (Fig. S1).

### Antibacterial Activity

Minimal inhibitory concentrations (MIC) were determined in M9 medium supplemented with 0.2% Casamino acids, 0.2% glucose, 1 mM MgSO_4_ and 1 µg/mL vitamin B1. 10 µL of double dilutions from a 1 mg/mL pyochelin solution were spotted on M9 agar plates and a lawn of the corresponding strain was overlaid. The maximum dilution that showed a zone of clearing was recorded as the MIC. FeCl_3_, ascorbic acid, citrate, enterobactin, were used at concentrations of 100 µM,1 mM, 50 µM, and 1 µM, respectively. Antibacterial activity of yersiniabacin (EMC Microcollections) was evaluated following the same procedure as for pyochelin.

### Purification of Enterobactin

Pure enterobactin was obtained from a ME9062 BW25113 culture supernatant following the protocol described by Winkelmann *et al*, 1994 [43]. Briefly, cells were grown for 20 hours at 37°C in M9 supplemented with 0.2% Casamino acids, 0.2% glucose, 1 mM MgSO_4_ and 1 µg/mL vitamin B1. The cell free supernatant was acidified to pH 2 using HCl, and enterobactin was extracted once with an equal volume of ethyl acetate. The extract was dried *in vacuo* and resuspended in methanol. Enterobactin was further purified by HPLC using a C18 Phenomenex Luna column (4.6×10 mm, 5 micron) and a gradient of 10 to 50% acetonitrile in water containing 0.1% trifluoracetic acid at a flow rate of 1 mL/min. The chromatographic profile obtained was similar to that reported by Winkelmann *et al* [43] (Fig. S2). The peak corresponding to enterobactin was corroborated using an enterobactin standard (EMC Microcollections). Enterobactin concentration was determined using the molar extinction coefficient (ε_319 nm_:11,200) [44].

Pure enterobactin was used in pyochelin MIC determinations by supplementing M9 plates with 1 µM enterobactin. Enterobactin was also used in ROS determinations at a concentration of 1 µM.

### Measurement of Reactive Oxygen Species

To determine the level of reactive oxygen species (ROS), exponentially growing cells in M9 minimal medium, were washed and resuspended in 50 mM sodium phosphate buffer, pH 7 at a final OD_600nm_ = 0.5. Then 2,7-dichlorofluorescein diacetate (H_2_DCFDA, the oxidation-sensitive probe dissolved in dimethyl sulfoxide) was added at a final concentration of 10 µM and incubated for 30 min [28]. After incubation, the cells were washed, resuspended and sonicated in the same buffer. Fluorescence intensity was measured using a Perkin Elmer LS55 spectrofluorometer (excitation λ, 490 nm; emission λ, 519 nm). Results are expressed as relative fluorescence to that of the control.

## Supporting Information

Figure S1 **Identification of pyochelin as the antibiotic compound produced by** ***P. aeruginosa*** **.** (A) HPLC-MS analysis of authentic pyochelin (black trace), the active antibiotic fraction from *P. aeruginosa* (red trace), and the *pchA* mutant of *P. aeruginosa* (blue trace), which does not display antibiotic activity. Note that pyochelin is isolated as two diastereomers, which correspond to the major peaks (black and red traces) observed with retention times of 12.8 and 13.8 min. (B) UV-visible spectra of the two diastereomeric peaks in authentic pyochelin (black and gray traces) and in the active antibiotic fraction isolated from *P. aeruginosa* (red and orange traces). The black and gray spectra are obtained for the peaks at 12.8 and 13.8 min, respectively, from authentic pyochelin. The red and orange spectra are obtained for the peaks at 12.8 and 13.8 min, respectively, from the active *P. aeruginosa* fraction. (C–F) Positive-ion mode mass spectra of the two diastereomeric peaks in authentic pyochelin (C, D) and in the antibiotic fraction isolated from *P. aeruginosa* (E, F). The mass spectra for the peaks at 12.8 and 13.8 min in authentic pyochelin are shown in panels (C) and (D), respectively. The mass spectra for the peaks at 12.8 and 13.8 min in the active *P. aeruginosa* fraction are shown in panels (E) and (F), respectively. In each case, the inset corresponds to a magnified view of the major mass ion. In all four cases [M+H]^+^observed = 325.1. For pyochelin, [M+H]^+^calculated = 325.1. Together, the identical retention times, UV-visible spectra, and mass spectra show that the active antibiotic fraction from *P. aeruginosa* is pyochelin.
(TIF)

Figure S2 **Enterobactin purification.** HPLC analysis of ethyl acetate extracts from wild type (red trace) and *E. coli entE* (blue trace) culture supernatants. The chromatogram obtained for the wild type strain, shows the two characteristic major peaks described by Winkelmann *et al* [43] (inset), corresponding to enterobactin (E) and the monomer involved in enterobactin synthesis, dihydroxybenzoyl serine (M). The peak corresponding to enterobactin displayed the same retention time as the enterobactin standard (black trace) and both enterobactin solutions showed a protective activity against pyochelin toxicity. The two major peaks (E and M) are absent in the chromatogram profile for the *entE* mutant strain (blue trace) and no collected fraction showed protective activity as it was expected.
(TIF)
