# Supplementary figures and images for: Catecholate Siderophores Protect Bacteria from Pyochelin Toxicity
Source: PLoS One. 2012 Oct 5;7(10):e46754. doi: 10.1371/journal.pone.0046754 (PMC3465284; doi:10.1371/journal.pone.0046754)

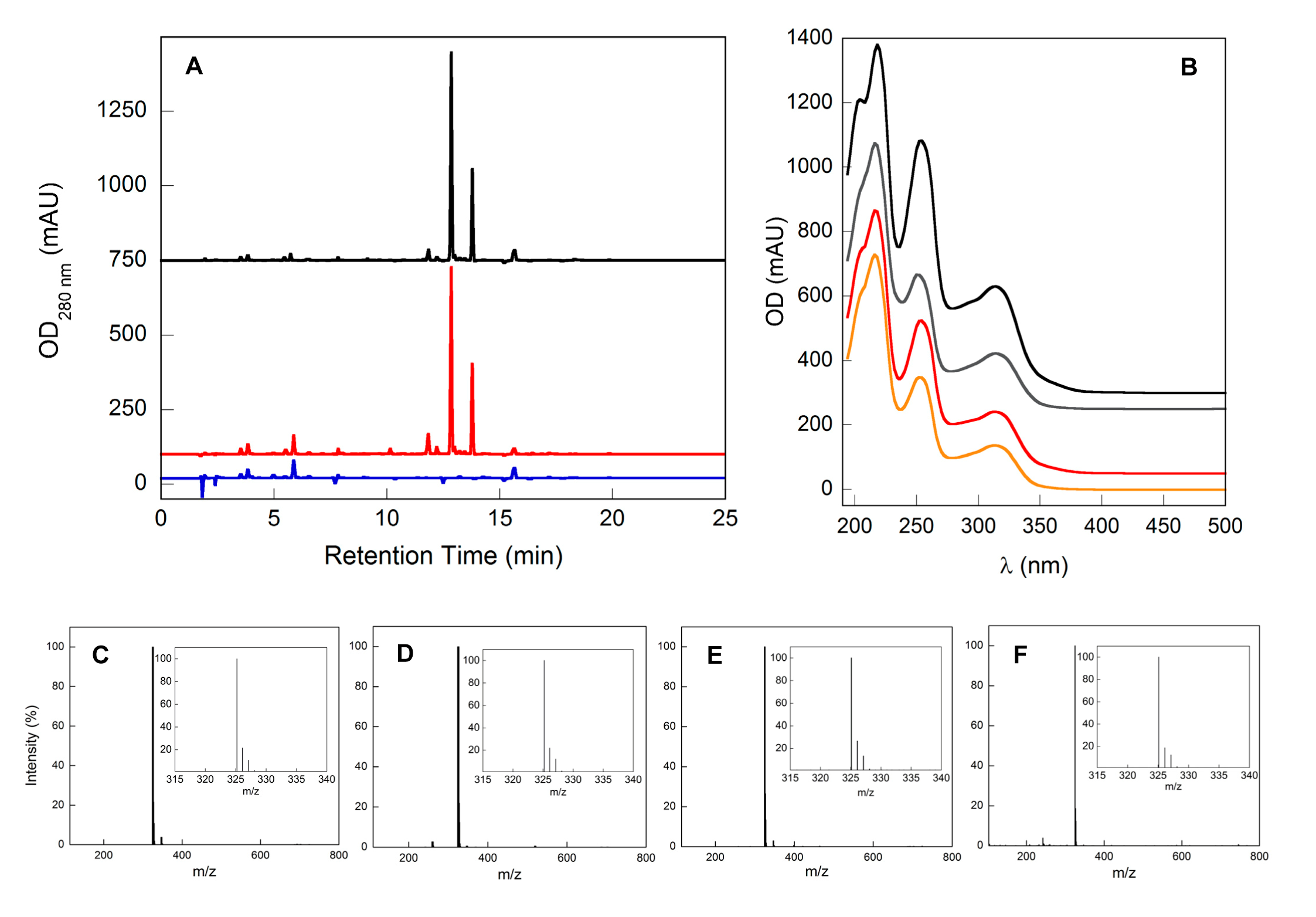

Supplement: Figure S1 — Identification of pyochelin as the antibiotic compound produced by P. aeruginosa . (A) HPLC-MS analysis of authentic pyochelin (black trace), the active antibiotic fraction from P. aeruginosa (red trace), and the pchA mutant of P. aeruginosa (blue trace), which does not display antibiotic activity. Note that pyochelin is isolated as two diastereomers, which correspond to the major peaks (black and red traces) observed with retention times of 12.8 and 13.8 min. (B) UV-visible spectra of the two diastereomeric peaks in authentic pyochelin (black and gray traces) and in the active antibiotic fraction isolated from P. aeruginosa (red and orange traces). The black and gray spectra are obtained for the peaks at 12.8 and 13.8 min, respectively, from authentic pyochelin. The red and orange spectra are obtained for the peaks at 12.8 and 13.8 min, respectively, from the active P. aeruginosa fraction. (C–F) Positive-ion mode mass spectra of the two diastereomeric peaks in authentic pyochelin (C, D) and in the antibiotic fraction isolated from P. aeruginosa (E, F). The mass spectra for the peaks at 12.8 and 13.8 min in authentic pyochelin are shown in panels (C) and (D), respectively. The mass spectra for the peaks at 12.8 and 13.8 min in the active P. aeruginosa fraction are shown in panels (E) and (F), respectively. In each case, the inset corresponds to a magnified view of the major mass ion. In all four cases [M+H]+observed = 325.1. For pyochelin, [M+H]+calculated = 325.1. Together, the identical retention times, UV-visible spectra, and mass spectra show that the active antibiotic fraction from P. aeruginosa is pyochelin. (TIF) [file pone.0046754.s001.tif]

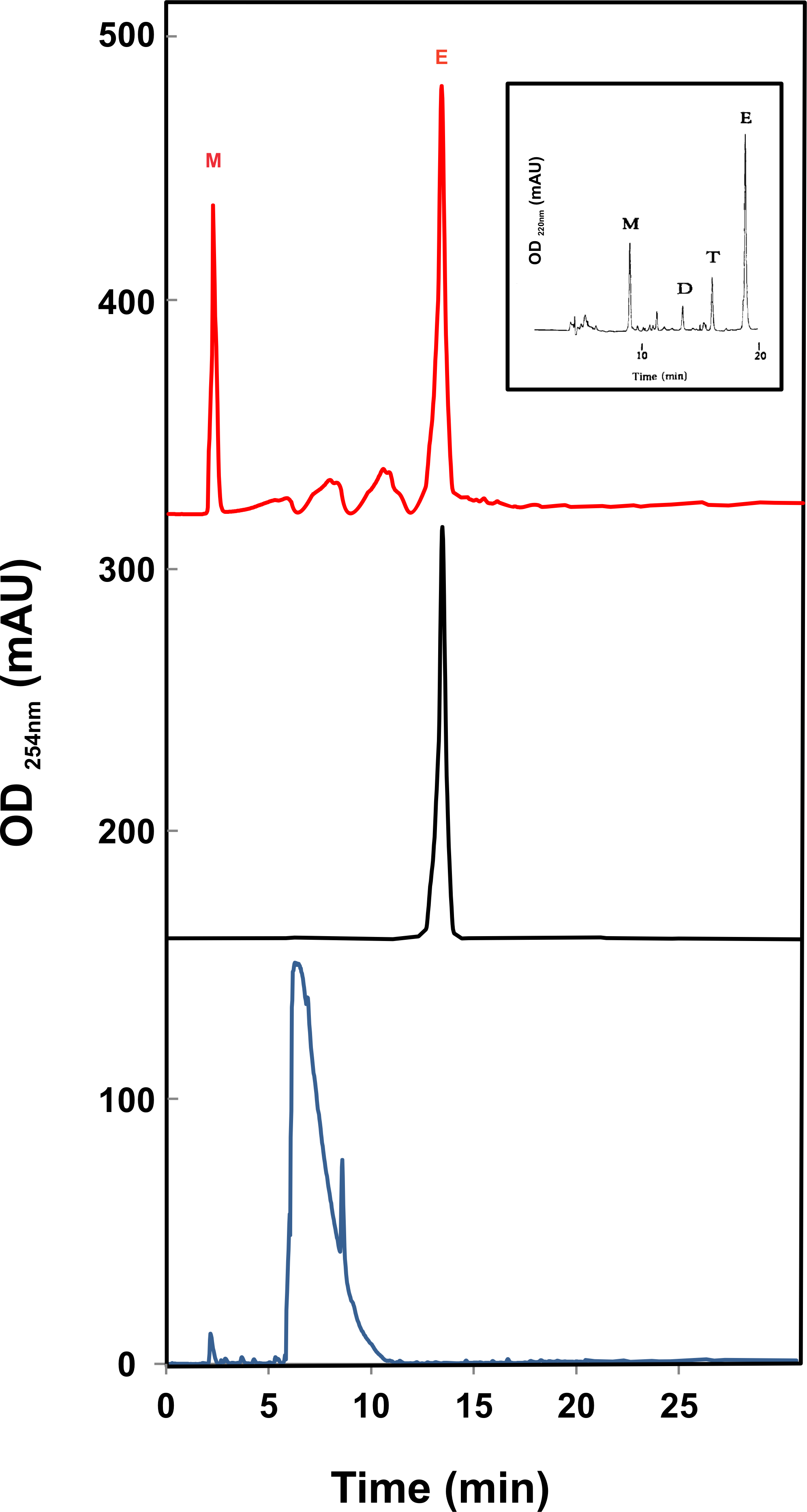

Supplement: Figure S2 — Enterobactin purification. HPLC analysis of ethyl acetate extracts from wild type (red trace) and E. coli entE (blue trace) culture supernatants. The chromatogram obtained for the wild type strain, shows the two characteristic major peaks described by Winkelmann et al [43] (inset), corresponding to enterobactin (E) and the monomer involved in enterobactin synthesis, dihydroxybenzoyl serine (M). The peak corresponding to enterobactin displayed the same retention time as the enterobactin standard (black trace) and both enterobactin solutions showed a protective activity against pyochelin toxicity. The two major peaks (E and M) are absent in the chromatogram profile for the entE mutant strain (blue trace) and no collected fraction showed protective activity as it was expected. (TIF) [file pone.0046754.s002.tif]
